# Frond Optical Properties of the Fern *Phyllitis scolopendrium* Depend on Light Conditions in the Habitat

**DOI:** 10.3390/plants9101254

**Published:** 2020-09-23

**Authors:** Mateja Grašič, Tjaša Sovdat, Alenka Gaberščik

**Affiliations:** Department of Biology, Biotechnical Faculty, University of Ljubljana, Večna pot 111, SI-1000 Ljubljana, Slovenia; tjasa.sovdat@gmail.com (T.S.); alenka.gaberscik@bf.uni-lj.si (A.G.)

**Keywords:** hart’s-tongue fern, *Phyllitis scolopendrium*, reflectance, transmittance, radiation gradient

## Abstract

Ferns display an elevated degree of phenotypic plasticity to changes in irradiance levels; however, only a few reports deal with their response to different light conditions. To get an insight into the extent of phenotypic plasticity of the fern *Phyllitis scolopendrium*, thriving in a forested area along a radiation gradient at the entrance of a cave, we examined selected biochemical, morphological, and physiological frond traits of the ferns from three different habitats. Sampling was performed two times during the vegetation season, in April and June. We also measured frond optical properties to point out the differences in leaf/light interactions between different plant samples. According to frond size, the middle habitat, receiving 125 µmol m^−2^s^−1^ of photosynthetically active radiation at both sampling times, appeared to be the most favourable. The production of UV-absorbing substances was highest in the habitat with the lowest radiation level. At the beginning of the season, the level of photosynthetic pigments in this habitat was the same as in the other habitats, while it was significantly lower in June when the tree canopy was closed. Frond reflectance was similar when comparing habitats and different sampling times. The most significant differences were obtained in the UV-A and near-infrared regions. The reflectance spectra depended mainly on frond biochemical properties, which altogether explained 54% (*p* ≤ 0.05) of the spectra variability. Frond transmittance depended on both, morphological parameters, explaining 51% (*p* ≤ 0.05), and frond biochemistry, explaining 73% (*p* ≤ 0.05) of the spectra variability. *P. scolopendrium* was revealed to be highly plastic regarding light conditions. The shapes of the frond reflectance and transmittance optical curves were similar to those typical of leaves of seed plants. The fronds exhibited high morphological plasticity when comparing different habitats. However, their biochemical and optical traits differed more between the two sampling times than between the habitats.

## 1. Introduction

Radiation environment may vary in time and space [1]. Changes in the radiation environment are a consequence of annual and diurnal radiation rhythms, but also occur due to biotic changes in different ecosystems or due to specific geological features of the landscape, such as steep slopes and depressions or entrances to caves. At cave entrances, there is a pronounced gradient of environmental conditions not only regarding light, but also regarding temperature and relative humidity [2]. Such a habitat gradient may host a variety of plant species, including ferns with high phenotypic plasticity [3,4]. Among others, ecosystems with pronounced changes in the radiation regime occur in temperate deciduous forests. There, radiation level gradually declines due to development of leaves in the tree canopy, which, in turn, affects plants growing in the understorey [5]. At the beginning of the vegetation season in spring, these plants may be exposed to direct sunlight, while later on, when the tree canopy is closed, they experience a significant decrease in the amount of radiation [6]. One of the most important abilities of these plants is the acclimation of their leaf optical properties and photosynthesis at the pigment level to these specific light conditions [7,8].

The interactions between solar radiation and plants are very complex, depending on the species’ biochemical and structural leaf traits. These are a consequence of the species’ genetic potential and specific environmental conditions in the habitat of a plant [9]. All the diverse leaf functional traits that develop under different radiation conditions optimise the harvesting of solar energy while also preventing damage due to the potentially harmful effects of photons [10]. Optical properties, which are a result of these traits, thus enable optimised leaf function [11]. The importance of specific traits in explaining leaf optical properties differs significantly among plant species [12,13,14,15], and, in many cases, also within a species [12,16]. The light reflected from leaves presents a kind of leaf spectral signature that may provide information about leaf biochemical and morphological traits [13,17,18,19,20] and also about its nutrient and water status [21,22,23]. In addition, leaf reflectance can contribute to the understanding of photosynthesis and leaf energy balance [24,25]. In comparison to spermatophytes, photosynthetic tissues of mosses and ferns possess specific anatomical and biochemical adaptations that might lead to differences in optical properties [11].

*Phyllitis scolopendrium* is a fern species that thrives at moist sites, such as wooded limestone ravines, steep north-facing slopes, and cave entrances, where it can colonise sites along a gradient of environmental conditions, including light [26,27], as also shown for many other ferns [28]. An important advantage of ferns in comparison to spermatophytes is their ability to photosynthesise under low light, which extends their potential habitat range [29]. This ability is not always the same, since it was shown that ferns might respond differently to different light levels in different phenological phases [30]. In spite of the great phenotypic plasticity of ferns regarding the radiation environment, the reports analysing their response to different light conditions are still scarce [31]. In the present study, we examined selected biochemical, morphological, and physiological frond traits of the fern *P. scolopendrium*, thriving in a forested area along a radiation gradient at the entrance of a cave, and measured their optical properties in order to point out the differences among plant specimens from the different habitats, sampled at different times during the vegetation season.

## 2. Materials and Methods

### 2.1. Studied Plant Species

Hart’s-tongue fern, *Phyllitis* (syn. *Asplenium*) *scolopendrium* (L.) Newm., is a perennial, rhizomatous, evergreen fern belonging to the Aspleniaceae family [32]. It is also a calciphile species, requiring habitats that are more or less permanently moist [29]. It has erect, tongue-shaped, leathery leaves called fronds, which may have wavy edges. The species has two varieties, *A. scolopendrium* L. var. *scolopendrium*, which is distributed broadly throughout Europe and Asia, and a rare tetraploid taxon *A. scolopendrium* L. var. *americanum* (Fern.) Kartesz and Gandhi that can be found in the eastern United States, Mexico, and Canada [33]. Cold winters may affect population growth rates and, thus, fern survival [34].

### 2.2. Site Description and Sampling

Experimental plants were sampled under the Little Natural Bridge near the entrance of the Zelške jame cave system in the Rakov Škocjan valley (45°47′30″ N, 14°18′22″ E; 575 m a.s.l.). Rakov Škocjan is a karst valley located at the northern foot of the Javorniki hills.

**Figure 1:**
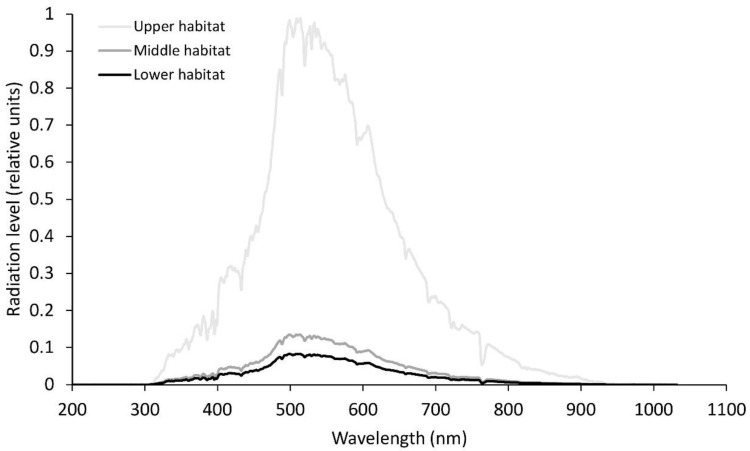
Relative solar radiation levels at noon-time for the different wavelengths for the three habitats in April. Average photosynthetically active radiation in the upper habitat, 800 µmol m^−2^s^−1^; middle habitat, 125 µmol m^−2^s^−1^; lower habitat, 8 µmol m^−2^s^−1^.

**Figure 2:**
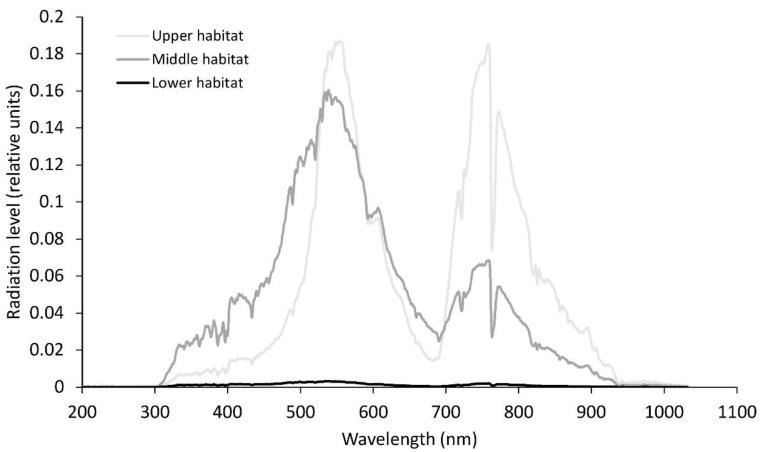
Relative solar radiation levels at noon-time for the different wavelengths for the three habitats in June. Average photosynthetically active radiation in the upper habitat, 158 µmol m^−2^s^−1^; middle habitat, 125 µmol m^−2^s^−1^; lower habitat, 2 µmol m^−2^s^−1^.

Sampling was performed twice during the vegetation season, namely, in April and June. Fronds sampled in April had developed in the previous season, while those sampled in June were fully developed fronds from the current season. Each time, ten samples were collected from three different habitats with varying light conditions. According to the distance of the habitats from the entrance of the cave system, these habitats were determined as (1) the upper habitat, which is the farthest from the cave system in the *Omphalodo-Fagetum sylvaticae* forest, (2) the middle habitat with somewhat lower light levels, and (3) the lower habitat with poor light conditions (Figure 1 and Figure 2) in the vicinity of the entrance to the cave system.

At the time when the tree canopy was closed, average mid-day temperature and relative humidity at the plant level were rather uniform, namely, 20.5 ± 0.3 °C and 76 ± 4% for the upper habitat, 21.3 ± 0.4 °C and 71 ± 4% for the middle habitat, and 19.6 ± 0.5 °C and 76 ± 3% for the lower habitat.

### 2.3. Relative Solar Radiation Level Measurements

Photosynthetically active radiation (PAR) was measured with a data logger (LI-1000; LI-COR, Inc., Lincoln, NE, USA) and a quantum sensor (LI-190SA; LI-COR, Inc., Lincoln, NE, USA). The radiation spectra were measured on the day of sampling in April and June using a portable spectrometer (Jaz Modular Optical Sensing Suite; Ocean Optics, Inc., Dunedin, FL, USA). A white reference panel (Spectralon; Labsphere, North Sutton, NH, USA) was used for calibration of the spectrometer to 100% reflectance prior to measurement. Ten measurements of PAR and ten measurements of solar radiation spectra from 200 to 1100 nm were performed at the plant level for each of the three habitats.

### 2.4. Biochemical Frond Traits

All of the biochemical analyses were conducted on vital, fully developed fronds. Chlorophyll *a*, chlorophyll *b*, and carotenoid contents were determined as described by Lichtenthaler and Buschmann [35,36]. Extract absorbance levels were measured at 470, 645, and 662 nm using a UV–VIS spectrometer (Lambda 25; Perkin-Elmer, Norwalk, CT, USA). The contents of chlorophylls and carotenoids were expressed per sample area (mg dm^−2^). Anthocyanin contents were determined according to Drumm and Mohr [37], with extract absorbance levels measured at 530 nm and the contents calculated per sample area (relative units per cm^−2^). We also measured the contents of the total methanol-soluble UV-B- and UV-A-absorbing substances, as proposed by Caldwell [38]. The absorbance levels of the extracts were measured in the spectral ranges of 280 to 319 nm for the UV-B-absorbing substances, and 320 to 400 nm for the UV-A-absorbing substances. The extinction values were integrated for each of the two UV regions and expressed in relative units per sample area.

### 2.5. Morphological Frond Traits

Morphological traits were studied on transverse frond sections, which were analysed under a light microscope (CX41; Olympus, Tokyo, Japan) equipped with a digital camera (XC30; Olympus, Tokyo, Japan) and CellSens software (Olympus, Tokyo, Japan). 100× magnification was used to measure thicknesses of the fronds, mesophyll, epidermis, and cuticle, while 400× magnification was used to determine stomata length and density. The latter two parameters were studied only for the lower frond surface. All of the measurements were performed on the central parts of vital and fully developed fronds.

### 2.6. Physiological Frond Traits

A portable chlorophyll fluorometer (PAM-2100; Heinz Walz GmbH, Effeltrich, Bavaria, Germany) was used for measurements of the potential and effective photochemical efficiencies of the photosystem (PS) II, which were evaluated according to Schreiber et al. [39]. Prior to the measurement of potential photochemical efficiency, the samples were kept in the dark for 20 min for dark adaptation. Stomatal conductance was recorded using a steady-state leaf porometer (Decagon Devices, Inc., Pullman, WA, USA), which measured the rate of water vapour diffusion via the leaf surfaces. All of the physiological parameters were measured in situ between 11:00 h and 14:00 h on vital and fully developed fronds of ten specimens from each habitat.

### 2.7. Optical Frond Traits

The optical traits of the fronds, namely, reflectance and transmittance, were measured from 300 to 800 nm on vital and fully developed fronds on the day they were collected. Measurements were performed using a Jaz Modular Optical Sensing Suite portable spectrometer (Ocean Optics, Inc., Dunedin, FL, USA) that was fitted with an ISP-30-6-R integrating sphere (Ocean Optics, Inc., Dunedin, FL, USA) and a QP600-1-SR-BX optical fibre (Ocean Optics, Inc., Dunedin, FL, USA).

Total adaxial reflectance spectra were recorded during illumination of fronds with a UV–VIS/near-infrared (NIR) light source (DH-2000; Ocean Optics, Inc., Dunedin, FL, USA). Before measurement, the spectrometer was calibrated to 100% reflectance using a white reference panel (Spectralon; Labsphere, North Sutton, NH, USA).

To measure the transmittance spectra, we first calibrated the spectrometer to 100% transmittance with a light beam that passed directly into the integrating sphere. Afterward, the integrating sphere was placed at the abaxial frond surface, while the UV–VIS/NIR light source illuminated the adaxial frond surface.

### 2.8. Statistical Analyses

Normal distributions of the data were evaluated using Shapiro–Wilk tests. Homogeneity of variance from the means was analysed using Levene’s tests. One-way analysis of variance (ANOVA) according to Duncan’s posthoc multiple range tests was used to assess differences between the six considered groups for each measured parameter. To investigate the relationships between the selected frond traits, we performed Pearson’s correlation analysis. IBM SPSS statistics 22.0 (IBM, Armonk, NY, United States) was used for these statistical calculations, with significance accepted at *p* ≤ 0.05. The figures for mean relative reflectance and transmittance spectra of the fronds from the three studied habitats, sampled at two different times during the vegetation season, were drawn in Microsoft Excel 2016 (Microsoft, Redmond, WA, USA).

Detrended correspondence analysis was used for the exploratory data analysis using the CANOCO for Windows 4.5 programme package. Due to the gradient lengths obtained (< 3 SD) [40], we used redundancy analysis to see how much of the variability of the frond spectra is explained by the biochemical and morphological parameters of these fronds. The significance of the effects of the variables was determined using Monte Carlo tests with 999 permutations. We used forward selection of the explanatory variables in order to avoid colinearity. All of the variables used in the analysis were standardised.

## 3. Results

### 3.1. Biochemical Frond Traits

Frond biochemical traits often showed significant differences (*p* ≤ 0.05) between the lower habitat and the remaining two habitats (Table 1). In April, this was seen for UV-absorbing substances, while in June, this was the case for all the studied pigments except for anthocyanins. In general, contents of the protective pigments (i.e., anthocyanins and UV-absorbing substances) were higher in the lower habitat and earlier in the vegetation season. Photosynthetic pigment contents (i.e., chlorophylls and carotenoids) were also higher in older leaves; however, they decreased with decreasing radiation level in the habitats.

### 3.2. Morphological Frond Traits

In both April and June, the lowest frond length and width were measured in the lower habitat, while significantly the widest and the longest fronds were found in the middle habitat (*p* ≤ 0.05; Table 1). No differences in frond width and length were observed with the progression of the vegetation season for both the lower and the upper habitat. Likewise, in the middle habitat, frond width also did not show any changes from April to June.

Frond and mesophyll thickness was generally higher in April. However, for both parameters, only the lower habitat in June showed significantly lower values compared to all the other groups. No significant differences were found for upper epidermis thickness. Lower epidermis thickness was higher in April but did not differ significantly between the three habitats within each of the two months. In April, upper and lower cuticle thickness increased with habitat depth. For the lower cuticle, a significant difference in April was only seen between the upper and the lower habitat, while upper cuticle thickness in April was significantly lower in the upper habitat compared to the middle and lower habitats. In June, the upper and lower cuticle thickness did not differ significantly between the three habitats. When comparing differences in cuticle thickness for fronds from the same habitat between the two months, only upper cuticle thickness in the lower habitat showed a significant difference between April and June.

Stomata density and length generally decreased with habitat depth, with the exception of stomata length in April, which showed an increase. These two parameters mostly did not differ between habitats within the same month, except for April, which showed significantly lower stomata density in the lower habitat in comparison to the other two habitats.

### 3.3. Physiological Frond Traits

The measured physiological frond parameters did not show significant differences between the three habitats in June (*p* ≤ 0.05; Table 1). In April, on the other hand, there was an increase in both effective and potential photochemical efficiency and a decrease in stomatal conductance with habitat depth. Potential photochemical efficiency and stomatal conductance for fronds from the lower habitat in April differed significantly from those for the upper and middle habitats, whereas effective photochemical efficiency in April showed significant differences between all the three habitat groups for this month. In general, effective and potential photochemical efficiency was much higher for all the three habitats in June and for the lower habitat in April than for the upper and middle habitats in April.

### 3.4. Optical Frond Traits

**Figure 3:**
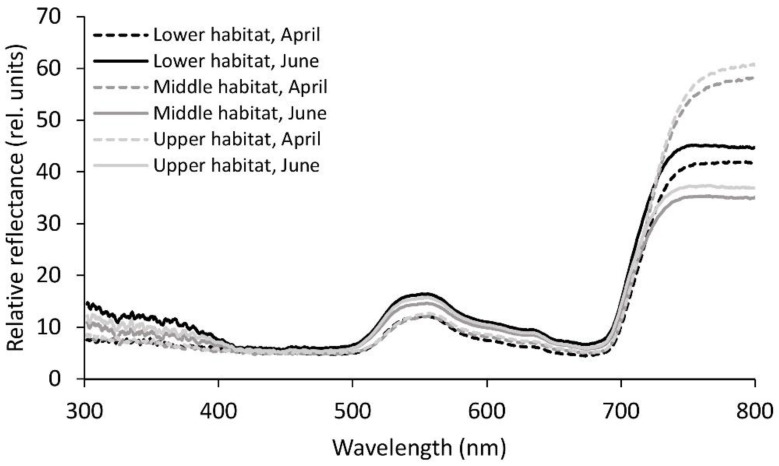
Mean relative reflectance spectra from 300 to 800 nm for the *Phyllitis scolopendrium* fronds from the three different habitats, sampled in April and June. The data were smoothed using moving averages with a period of five consecutive measurements (n = 10).

The reflectance of the fronds was generally lower in April than in June, except for the significantly higher measured values in the NIR part of the spectrum for the upper and middle habitats in April (*p* ≤ 0.05; Table 2, Figure 3). Besides the NIR region, there were not many significant differences in reflectance between the three habitats in April, except for the significantly higher measured values in the upper habitat compared to the lower habitat in yellow and red. In June, the reflectance was more variable. For the UV-B region, the three habitat groups did not show any significant differences, whereas all the three habitat groups differed significantly from each other in the UV-A region of the spectrum. In all the regions of the spectrum from UV-A onwards, the reflectance was highest in the lower habitat and lowest in the middle habitat. For the violet, green, yellow, and NIR regions, the difference between the middle and lower habitats in reflectance was significant. In the blue and red regions, the lower habitat differed significantly from both the middle and upper habitats.

**Figure 4:**
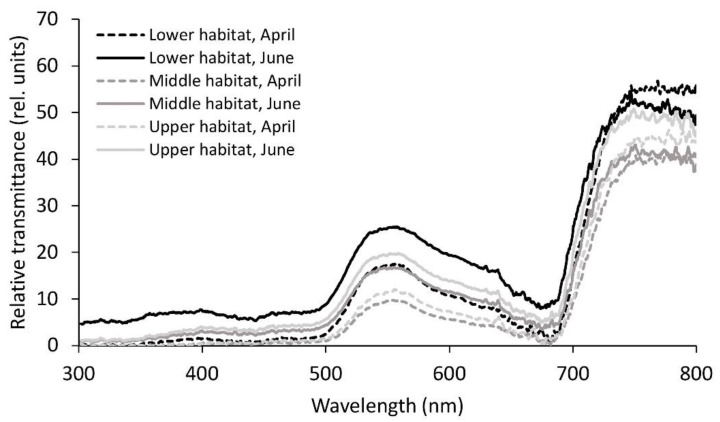
Mean relative transmittance spectra from 300 to 800 nm for the *Phyllitis scolopendrium* fronds from the three different habitats, sampled in April and June. The data were smoothed using moving averages with a period of five consecutive measurements (n = 10).

Similar to reflectance, transmittance was also lower in April compared to June, again with the exception of the NIR part of the spectrum, where transmittance in the lower habitat in April was the highest among all of the six studied groups (Table 2, Figure 4). In addition to the NIR region, transmittance in April only showed significant differences in green and yellow, being higher in the lower habitat compared to the remaining two habitats. In June, fronds from the lower habitat displayed significantly higher transmittance compared to the ones from the other two habitats in all the regions of the spectrum except NIR. NIR transmittance was also highest in the lower habitat; however, the difference was only significant towards the middle habitat.

### 3.5. Relationships between Selected Frond Parameters

When the data were combined for the two months, Pearson’s correlation analysis showed a significant negative correlation between frond thickness and frond optical properties (reflectance and transmittance) throughout the whole spectrum, except for the NIR region (*p* ≤ 0.05; Table 3). The same relationship with frond optical properties was also found to be significant for UV-B-absorbing substances, but not for UV-A-absorbing substances.

**Figure 5:**
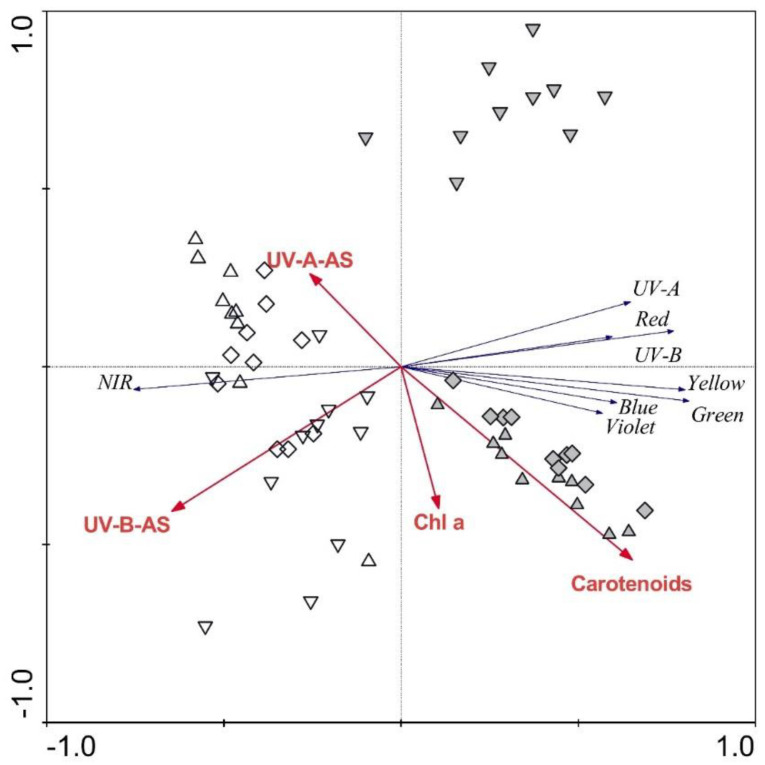
Redundancy analysis plot showing the strength of the associations between the frond traits and the regions of the reflectance spectra for *Phyllitis scolopendrium*. Up-triangles, specimens from the upper habitat; diamonds, specimens from the middle habitat; down-triangles, specimens from the lower habitat; white symbols, April samples; grey symbols, June samples; Chl a, chlorophyll *a*; AS, absorbing substances; NIR, near-infrared.

The RDA plot showing the strength of the associations between frond morphological parameters and frond reflectance spectra revealed the relatively low importance of frond morphology in explaining the reflectance spectra. Only frond thickness was significant (*p* ≤ 0.05), explaining 9% of the reflectance spectra variability. The RDA plot showing the strength of the associations between frond biochemical parameters and the regions of the reflectance spectra (Figure 5) revealed the high importance of frond biochemistry in explaining frond reflectance. When examining simple effects, carotenoids alone explained 23%, and UV-B-absorbing substances alone explained 22% of the spectra variability. When tested altogether, carotenoids and UV-B-absorbing substances explained 23% (*p* = 0.01) and 13% (*p* = 0.01), respectively, chlorophyll *a* explained 12% (*p* = 0.02), and UV-A-absorbing substances explained additional 6%. April samples are positioned in the left part of the plot, while June samples are mainly located in the right part of the plot.

**Figure 6:**
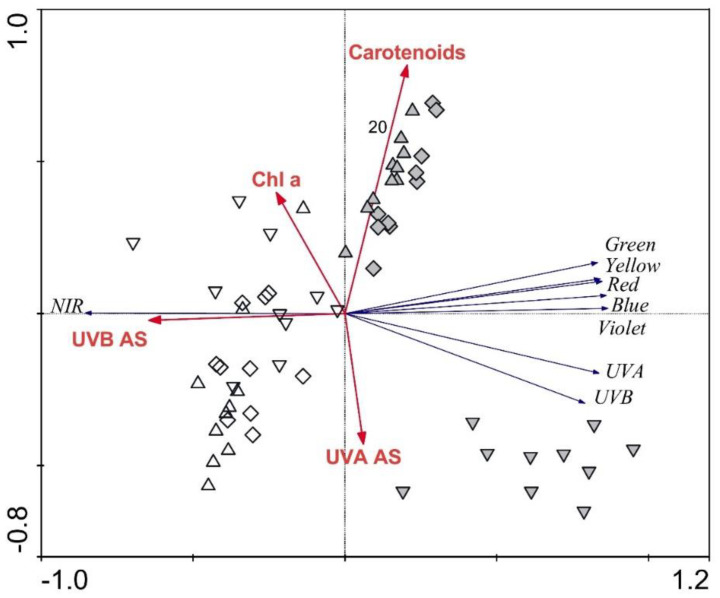
Redundancy analysis plot showing the strength of the associations between the frond traits and the regions of the transmittance spectra for *Phyllitis scolopendrium*. Up-triangles, specimens from the upper habitat; diamonds, specimens from the middle habitat; down-triangles, specimens from the lower habitat; white symbols, April samples; grey symbols, June samples; Chl a, chlorophyll *a*; AS, absorbing substances; NIR, near-infrared.

The RDA analysis displaying the strength of the associations between frond morphological parameters and frond transmittance revealed the high importance of frond morphology in explaining frond transmittance. The majority of the parameters were revealed to be significant, explaining 51% of the transmittance spectra variability. Again, the most influential parameter was frond thickness, explaining 29% (*p* = 0.001), while upper and lower epidermis, mesophyll, and lower cuticle thicknesses explained 5% (*p* ≤ 0.05) or 6% (*p* ≤ 0.05) each. The RDA plot showing the strength of the associations between frond biochemical parameters and the regions of the transmittance spectra revealed that UV-B- and UV-A-absorbing substances explained 30% (*p* = 0.001) of the spectra variability each, while carotenoids and chlorophyll *a* explained an additional 11% (*p* = 0.001) and 2% (*p* = 0.018), respectively. Altogether, the biochemical parameters explained 73% of the transmittance spectra variability (Figure 6). The three groups for April samples are somewhat overlapping, while June samples from the three different habitats are distributed in distinct groups. As for the reflectance spectra, the vectors for the UV and visible spectra oppose the NIR vector.

## 4. Discussion

Plant environment is very complex since it is affected by a variety of abiotic and biotic factors, among which light, as a primary source of energy, presents a crucial factor. Light conditions for experimental plants differed among habitats and regarding the time of the season, with the exception of the middle habitat, where the level of PAR was 125 µmol m^−2^s^−1^ at both sampling times. However, the values for temperature and relative humidity were more uniform. These uniform conditions, especially during canopy closure, are a consequence of a specific microclimate that develops within the vegetation layer [9]. At the beginning of the season, before canopy closure, plants from the upper habitat experienced much higher solar radiation levels in comparison to the levels later in the season, when the forest canopy absorbed much of the red light. The differences in the quality and quantity of available radiation affected frond size, which was largest in the middle habitat. In the case of the fern *Platycerium bifurcatum*, smaller frond blade size was related to the increased R/FR (red/far-red) value, which is characteristic of high radiation environments [41]. In our study, the differences in red and far-red light were more pronounced at the beginning of the growing season; however, later on, the differences between the upper and middle habitats decreased, which was also reflected in smaller differences in frond size. A variable radiation regime affects numerous plant functions [1], including the synthesis of different pigments, as also revealed from our research. Oliwa et al. [41] analysed frond optical properties in *Platycerium bifurcatum* and determined a higher accumulation of carotenoids and anthocyanins in the fronds of plants grown under lower R/FR ratios. However, this was not the case in our study. Frond pigment contents of the *P. scolopendrium* samples from April were comparable for the three habitats; an exception was seen for the contents of UV-absorbing substances, which were significantly higher for the lower habitat. For the second sampling, the contents of photosynthetic pigments increased, with the exception of the lower habitat, where we observed a decrease. The increase in photosynthetic pigments was more pronounced for chlorophyll *b*, which resulted in a lower chlorophyll *a* to *b* ratio. This change in the chlorophyll *a* to *b* ratio is acclimation to an altered radiation regime [42]. On the contrary, the production of UV-absorbing substances decreased, but not for the lower habitat. This higher level of UV-absorbing substances in the samples from the lower habitat was against expectations, as this habitat was exposed to the lowest radiation level. The production of UV-absorbing substances, which are represented by different flavonoids, is usually triggered by high light intensity, including UV radiation [43,44]. In some higher plants, it has been shown that UV-absorbing substances might also be induced by low temperatures in the absence of UV-B radiation [45]. However, this was probably not the main cause in our study with the fern *P. scolopendrium*. Flavonoids are very important substances for plants as they are involved in the interactions of plants with other organisms and mitigate different environmental stresses, including protection against high radiation levels, which results from their antioxidative potential [46]. Shade plants with a large and thin leaf blade may produce different kaempferol and/or apigenin derivatives [47], as is also the case in the species studied here, *P. scolopendrium* [48,49]. Kaempferols absorb radiation in the UV and blue regions of the spectrum, showing lower absorption in the UV-B region in comparison to the UV-A region, with peaks at around 260 and 375 nm, respectively [50]. In spite of that, these kaempferols are less efficient in protection against UV radiation but present efficient protection against a variety of pathogens [46]. Therefore, high contents of UV-absorbing substances might also be expected in fronds that overwintered and were sampled in April, as well as in fronds that developed under low light conditions, as was the case for the lower habitat in our study. The substances, which absorb in the UV-B region of the spectrum, were negatively related to frond reflectance and transmittance, while no correlation was obtained for the substances that absorb in the UV-A region, which also include kaempferols.

The chlorophyll fluorescence measurements in our study may indicate the presence of stress in plants [51,52]. For *P. scolopendrium* from the upper and middle habitats, effective photochemical efficiency (yield) was negatively affected in spring prior to the unfolding of leaves in the tree canopy. On the other hand, potential photochemical efficiency (Fv/Fm) was relatively high [39]. The Fv/Fm values were comparable to those measured in the leaves of some rainforest species grown under less than 200 µmol m^−2^, which ranged from 0.7 to 0.8 [53]. Therefore, the difference between both measured chlorophyll fluorescence parameters only reveals transient stress. This transient stress was probably due to a mid-day high radiation level and, at the same time, fully open stomata, as indicated by high stomatal conductance, which can result in a decrease in frond water potential [9]. The study by Cardoso et al. [54] showed that in ferns, the stomata responded directly to the changes in leaf water status and not to the metabolic processes related to the production of abscisic acid (ABA) [55]. The stomata of *P. scolopendrium* consist of guard cells that contain more chloroplasts than the guard cells of angiosperms, and they can open widely even under conditions of limited photosynthetic carbon dioxide fixation [56]. In addition, different species may respond differently to a changed radiation regime, as shown for the genus *Asplenium*. Namely, *A. ceterach* seems to be more efficient under full sunlight conditions in comparison to *A. trichomanes*, where a decrease in maximum photochemical efficiency and slower energy flow through PSII were observed [31].

Changes in frond optical properties during the season depend on changes in pigment contents, anatomy, and senescence processes [57]. The reflectance curves of different specimens of the studied plants were very similar in April; however, in June, they differed significantly in some regions, which might be related to the radiation regime. The differences in the UV-A and NIR regions were the most evident. The differences in transmittance were generally much more pronounced in comparison to reflectance. Moreover, they increased with time. Using RDA, we explained the greatest share of the reflectance and transmittance spectra variability with UV-absorbing substances, while a minor part was also explained with chlorophyll and carotenoid contents. It was shown before that the reflectance of light in the visible part of the spectrum was related to the contents of photosynthetic pigments, which efficiently harvest more than 90% of incoming radiation [58]. An important role in shaping leaf optical properties was also attributed to leaf thickness, as shown by many other studies [12]. The shape of the frond reflectance spectra was similar to those typical of leaves of seed plants [9].

## 5. Conclusions

According to frond size, the middle habitat revealed to be the most favourable. The production of UV-A-absorbing substances was higher in the overwintered fronds in comparison to the fronds of the current season. However, when comparing different habitats, it was highest in the habitat with the lowest radiation level. An increase in photosynthetic pigments and a decrease in UV-absorbing substances during the season in the upper and middle habitats, and just the opposite response in the lower habitat, were likely a consequence of the trade-off between input in plant assimilation and plant protection. Frond spectral signatures (reflectance) differed less between the habitats than between the two different sampling times and were mostly related to frond biochemical properties, which should be taken into account when monitoring this species with the remote sensing technique. On the other hand, frond transmittance depended on both, frond morphology and biochemistry.

We can conclude that *P. scolopendrium* exhibits a highly plastic response regarding the examined physiological, biochemical, and morphological traits, which enables plants to use the advantage of specific environmental conditions. The higher production of UV-A-absorbing substances in this species, which were produced under less favourable conditions, is likely in the function of protection against pathogens that could develop under such conditions in winter and in a low radiation environment. This deserves further investigation, since it presents an important aspect of the future success of this fern species.

## Figures and Tables

**Table 1:** Biochemical, morphological, and physiological traits of the *Phyllitis scolopendrium* fronds from the three habitats with different light conditions, sampled in April and June.

Frond Traits	Units	April			June		
		Upper Habitat	Middle Habitat	Lower Habitat	Upper Habitat	Middle Habitat	Lower Habitat
**Biochemical**							
Chlorophyll *a*	mg dm^−2^	3.61 ± 0.96 ^bc^	3.05 ± 1.27 ^ab^	3.39 ± 0.85 ^bc^	4.18 ± 0.73 ^c^	4.01 ± 0.87 ^c^	2.28 ± 0.46 ^a^
Chlorophyll *b*	mg dm^−2^	3.55 ± 1.05 ^b^	2.78 ± 0.99 ^ab^	2.66 ± 1.13 ^ab^	4.96 ± 1.15 ^c^	4.63 ± 1.42 ^c^	2.18 ± 0.58 ^a^
Carotenoids	mg dm^−2^	0.60 ± 0.14 ^a^	0.56 ± 0.31 ^a^	0.63 ± 0.13 ^a^	2.14 ± 0.40 ^b^	2.06 ± 0.51 ^b^	0.34 ± 0.04 ^a^
Anthocyanins	au cm^−2^	0.58 ± 0.08 ^ab^	0.61 ± 0.11 ^c^	0.65 ± 0.15 ^c^	0.46 ± 0.07 ^a^	0.49 ± 0.12 ^ab^	0.58 ± 0.18 ^ab^
UV-B-AS	au cm^−2^	3.16 ± 0.93 ^b^	3.06 ± 0.51 ^b^	4.42 ± 1.06 ^c^	1.64 ± 0.16 ^a^	1.70 ± 0.32 ^a^	1.74 ± 0.41 ^a^
UV-A-AS	au cm^−2^	4.33 ± 1.65 ^b^	4.24 ± 0.74 ^b^	6.51 ± 1.24 ^c^	2.07 ± 0.27 ^a^	2.50 ± 0.60 ^a^	6.34 ± 1.31 ^c^
**Morphological**							
Frond length	cm	23.5 ± 4.9 ^b^	40.9 ± 8.3 ^d^	14.6 ± 6.9 ^a^	22.8 ± 3.9 ^b^	29.4 ± 2.3 ^c^	16.4 ± 2.4 ^a^
Frond width	cm	4.8 ± 1.0 ^a^	6.3 ± 0.7 ^b^	4.5 ± 0.9 ^a^	5.1 ± 0.5 ^a^	6.7 ± 0.8 ^b^	5.0 ± 0.8 ^a^
Frond thickness	µm	327.5 ± 32.0 ^b^	343.2 ± 38.5 ^b^	340.7 ± 47.9 ^b^	313.4 ± 29.3 ^b^	309.5 ± 40.0 ^b^	240.2 ± 47.2 ^a^
Upper cuticle	µm	8.1 ± 2.3 ^a^	11.2 ± 5.9 ^b^	12.1 ± 3.2 ^b^	8.0 ± 1.7 ^a^	9.2 ± 1.8 ^ab^	7.0 ± 2.1 ^a^
Upper epidermis	µm	32.8 ± 2.9 ^a^	34.4 ± 6.0 ^a^	35.5 ± 7.3 ^a^	31.5 ± 6.3 ^a^	28.8 ± 6.0 ^a^	34.0 ± 11.4 ^a^
Mesophyll	µm	239.8 ± 29.3 ^b^	249.4 ± 34.2 ^b^	239.8 ± 43.4 ^b^	237.0 ± 26.8 ^b^	235.5 ± 38.1 ^b^	170.0 ± 46.2 ^a^
Lower epidermis	µm	33.5 ± 7.6 ^bc^	34.7 ± 6.5 ^c^	30.6 ± 8.7 ^abc^	28.5 ± 6.5 ^abc^	26.6 ± 6.6 ^ab^	25.6 ± 7.4 ^a^
Lower cuticle	µm	6.6 ± 1.5 ^a^	8.9 ± 4.7 ^ab^	9.5 ± 3.4 ^b^	6.8 ± 1.8 ^a^	6.5 ± 1.3 ^a^	7.2 ± 1.7 ^ab^
Stomata length	µm	56.4 ± 4.8 ^a^	59.7 ± 4.6 ^abc^	59.1 ± 4.2 ^ab^	64.6 ± 7.2 ^c^	63.6 ± 5.7 ^bc^	61.4 ± 3.8 ^abc^
Stomata density	cm^−2^	20 ± 3 ^c^	19 ± 3 ^bc^	11 ± 3 ^a^	18 ± 5 ^bc^	16 ± 3 ^b^	15.5 ± 2.3 ^b^
**Physiological**							
Stomatal conductance	mmol m^−2^ s^−1^	158.2 ± 59.3 ^c^	145.0 ± 37.9 ^c^	73.5 ± 15.7 ^b^	38.0 ± 24.5 ^a^	34.58 ± 6.49 ^a^	54.9 ± 9.5 ^ab^
Yield	au	0.28 ± 0.09 ^a^	0.46 ± 0.05 ^b^	0.76 ± 0.02 ^d^	0.63 ± 0.21 ^c^	0.70 ± 0.12 ^cd^	0.71 ± 0.05 ^cd^
Fv/Fm	au	0.70 ± 0.06 ^a^	0.73 ± 0.04 ^a^	0.80 ± 0.02 ^b^	0.81 ± 0.01 ^b^	0.79 ± 0.02 ^b^	0.78 ± 0.04 ^b^

Data are means ± SD (n = 10 for each column). Different superscript letters within each row indicate significant differences (*p* ≤ 0.05; Duncan tests). Yield, effective photochemical efficiency; Fv/Fm, potential photochemical efficiency; UV-B-AS, UV-B-absorbing substances; UV-A-AS, UV-A-absorbing substances; au, arbitrary units.

**Table 2:** Optical traits of the *Phyllitis scolopendrium* fronds from the three habitats with different light conditions, sampled in April and June.

Optical Frond Traits	Units	April			June		
		Upper Habitat	Middle Habitat	Lower Habitat	Upper Habitat	Middle Habitat	Lower Habitat
**Reflectance**	au						
UV-B		8.31 ± 0.26 ^a^	9.57 ± 4.98 ^ab^	7.66 ± 0.39 ^a^	11.74 ± 1.80 ^bc^	12.12 ± 5.09 ^bc^	13.68 ± 1.59 ^c^
UV-A		6.74 ± 0.25 ^a^	6.50 ± 0.26 ^a^	6.78 ± 0.32 ^a^	9.20 ± 1.51 ^c^	8.14 ± 1.16 ^b^	10.73 ± 1.36 ^d^
Violet		5.25 ± 0.24 ^ab^	5.08 ± 0.32 ^a^	5.27 ± 0.19 ^ab^	5.74 ± 0.93 ^bc^	5.54 ± 0.44 ^ab^	6.15 ± 0.67 ^c^
Blue		5.10 ± 0.29 ^ab^	4.93 ± 0.38 ^a^	4.91 ± 0.32 ^a^	5.55 ± 0.98 ^b^	5.52 ± 0.52 ^b^	6.10 ± 0.78 ^c^
Green		10.00 ± 0.58 ^a^	9.61 ± 1.62 ^a^	9.67 ± 0.63 ^a^	12.73 ± 1.43 ^bc^	11.94 ± 1.18 ^b^	13.37 ± 2.04 ^c^
Yellow		8.58 ± 0.71 ^b^	8.14 ± 1.44 ^ab^	7.48 ± 0.64 ^a^	10.46 ± 1.39 ^cd^	9.88 ± 1.03 ^c^	11.02 ± 1.50 ^d^
Red		6.78 ± 0.61 ^bc^	6.24 ± 0.84 ^ab^	5.62 ± 0.50 ^a^	8.18 ± 1.25 ^d^	7.44 ± 0.78 ^cd^	9.26 ± 1.09 ^e^
NIR		48.18 ± 1.44 ^c^	46.49 ± 2.26 ^c^	36.07 ± 2.24 ^ab^	33.85 ± 2.52 ^ab^	31.83 ± 3.44 ^a^	40.23 ± 15.77 ^b^
**Transmittance**	au						
UV-B		0.05 ± 0.06 ^a^	0.12 ± 0.11 ^a^	0.12 ± 0.11 ^a^	1.28 ± 1.31 ^a^	1.07 ± 0.70 ^a^	5.12 ± 2.85 ^b^
UV-A		0.06 ± 0.06 ^a^	0.15 ± 0.14 ^a^	0.70 ± 0.42 ^ab^	2.22 ± 1.92 ^c^	1.72 ± 0.96 ^bc^	6.35 ± 3.03 ^d^
Violet		0.63 ± 0.45 ^a^	0.50 ± 0.41 ^a^	1.06 ± 0.46 ^a^	3.59 ± 2.38 ^b^	2.71 ± 1.11 ^b^	6.34 ± 2.50 ^c^
Blue		1.07 ± 0.65 ^a^	0.77 ± 0.56 ^a^	1.67 ± 0.69 ^a^	4.49 ± 2.68 ^b^	3.42 ± 1.31 ^b^	7.32 ± 2.73 ^c^
Green		8.50 ± 2.37 ^a^	6.93 ± 2.30 ^a^	13.18 ± 3.58 ^b^	15.90 ± 5.47 ^b^	13.33 ± 4.17 ^b^	21.23 ± 5.50 ^c^
Yellow		7.22 ± 2.40 ^a^	5.67 ± 2.11 ^a^	10.77 ± 3.13 ^b^	13.91 ± 5.21 ^b^	11.51 ± 3.62 ^b^	19.49 ± 5.15 ^c^
Red		3.87 ± 1.57 ^a^	3.10 ± 1.54 ^a^	5.66 ± 1.84 ^ab^	9.20 ± 4.10 ^c^	7.19 ± 2.41 ^bc^	13.09 ± 4.10 ^d^
NIR		38.44 ± 6.88 ^ab^	34.49 ± 6.79 ^a^	48.19 ± 7.74 ^c^	44.53 ± 7.14 ^bc^	37.23 ± 10.99 ^ab^	47.42 ± 11.27 ^c^

Data are means ± SD (n = 10 for each column). Different superscript letters within each row indicate significant differences (*p* ≤ 0.05; Duncan tests). Reflectance and transmittance spectra represent means within 5-nm intervals (*p* ≤ 0.05, Duncan tests). NIR, near-infrared; au, arbitrary units.

**Table 3:** Pearson’s correlation coefficients for the *Phyllitis scolopendrium* fronds from the three habitats with different light conditions for the combined data from April and June.

**Reflectance**	**Frond Thickness**	**UV-B-Absorbing Substances**
UV-B	−0.38 **	−0.42 **
UV-A	−0.46 **	−0.54 **
Violet	−0.29 *	−0.35 **
Blue	−0.34 **	−0.41 **
Green	−0.46 **	−0.59 **
Yellow	−0.45 **	−0.61 **
Red	−0.51 **	−0.63 **
NIR	0.01	0.16
**Transmittance**	**Frond Thickness**	**UV-B-Absorbing Substances**
UV-B	−0.52 **	−0.41 **
UV-A	−0.52 **	−0.43 **
Violet	−0.50 **	−0.50 **
Blue	−0.49 **	−0.48 **
Green	−0.43 **	−0.27 *
Yellow	−0.45 **	−0.33 *
Red	−0.47 **	−0.40 **
NIR	−0.17	0.14

**, *p* ≤ 0.01; *, *p* ≤ 0.05; NIR, near-infrared.

## References

[1] Glime J.M., Glime J.M. (2017). Light: The shade plants. Bryophyte Ecology. Volume 1. Physiological Ecology.

[2] Romero A. (2009). Cave Biology.

[3] Pentecost A., Zhaohui Z. (2001). The distribution of plants in Scoska Cave, North Yorkshire, and their relationship to light intensity. Int. J. Speleol..

[4] Monro A.K., Bystriakova N., Fu L., Wen F., Wei Y. (2018). Discovery of a diverse cave flora in China. PLoS ONE.

[5] Chazdon R.L., Pearcy R.W. (1991). The importance of sunflecks for forest understory plants: Photosynthetic machinery appears adapted to brief, unpredictable periods of radiation. BioScience.

[6] Esteban R., Fernández-Marín B., Becerril J.M., García-Plazaola J.I. (2008). Photoprotective implications of leaf variegation in *E. dens-canis* L. and *P. officinalis* L.. J. Plant Physiol..

[7] Popović Z., Mijović A., Karadžić B., Mijatović M. (2006). Response of growth dynamics of two spring geophytes to light regime in a lime-beech forest. J. Integr. Plant Biol..

[8] Klančnik K., Levpušček M., Gaberščik A. (2016). Variegation and red abaxial epidermis define the leaf optical properties of *Cyclamen purpurascens*. Flora.

[9] Larcher W. (2003). Physiological Plant Ecology: Ecophysiology and Stress Physiology of Functional Groups.

[10] Gurevitch J., Scheiner S.M., Fox G.A. (2002). The Ecology of Plants.

[11] Ustin S.L., Jacquemoud S., Cavender-Bares J., Gamon J., Townsend P. (2020). How the optical properties of fronds modify the absorption and scattering of energy and enhance frond functionality. Remote Sensing of Plant Biodiversity.

[12] Klančnik K., Mlinar M., Gaberščik A. (2012). Heterophylly results in a variety of “spectral signatures” in aquatic plant species. Aquat. Bot..

[13] Klančnik K., Vogel-Mikuš K., Gaberščik A. (2014). Silicified structures affect leaf optical properties in grasses and sedge. J. Photochem. Photobiol. B.

[14] Klančnik K., Vogel-Mikuš K., Kelemen M., Vavpetič P., Pelicon P., Kump P., Jezeršek D., Gianoncelli A., Gaberščik A. (2014). Leaf optical properties are affected by the location and type of deposited biominerals. J. Photochem. Photobiol. B.

[15] Klančnik K., Iskra I., Gradinjan D., Gaberščik A. (2018). The quality and quantity of light in the water column are altered by the optical properties of natant plant species. Hydrobiologia.

[16] Lukeš P., Stenberg P., Rautiainen M., Mõttus M., Vanhatalo K.M. (2013). Optical properties of leaves and needles for boreal trees species in Europe. Remote Sens. Lett..

[17] Levizou E., Drilias P., Psaras G.K., Manetas Y. (2005). Nondestructive assessment of leaf chemistry and physiology through spectral reflectance measurements may be misleading when changes in trichome density co-occur. New Phytol..

[18] Klančnik K., Gaberščik A. (2016). Leaf spectral signatures differ in plant species colonizing habitats along a hydrological gradient. J. Plant Ecol..

[19] De Tomás Marín S., Novák M., Klančnik K., Gaberščik A. (2016). Spectral signatures of conifer needles mainly depend on their physical traits. Pol. J. Ecol..

[20] Grašič M., Sakovič T., Abram D., Vogel-Mikuš K., Gaberščik A. (2020). Do soil and leaf silicon content affect leaf functional traits in *Deschampsia caespitosa* from different habitats. Biol. Plant..

[21] Asner G.P., Martin R.E. (2008). Spectral and chemical analysis of tropical forests: Scaling from leaf to canopy levels. Remote Sens. Environ..

[22] Roelofsen H.D., van Bodegom P.M., Kooistra L., Witte J.P.M. (2014). Predicting leaf traits of herbaceous species from their spectral characteristics. Ecol. Evol..

[23] Serbin S.P., Singh A., McNeil B.E., Kingdon C.C., Townsend P.A. (2014). Spectroscopic determination of leaf morphological and biochemical traits for northern temperate and boreal tree species. Ecol. Appl..

[24] Ullah S., Schlerf M., Skidmore A.K., Hecker C. (2012). Identifying plant species using midwave infrared 2.5–6-μm and thermal infrared 8–14-μm emissivity spectra. Remote Sens. Environ..

[25] Noda H.M., Motohka T., Murakami K., Muraoka H., Nasahara K.N. (2013). Accurate measurement of optical properties of narrow leaves and conifer needles with a typical integrating sphere and spectroradiometer. Plant Cell Environ..

[26] Futyma R.P. (1980). The distribution and ecology of *Phyllitis scolopendrium* in Michigan. Am. Fern J..

[27] Martinčič A., Wraber T., Jogan N., Podobnik A., Turk B., Vreš B., Ravnik V., Frajman B., Strgulc Krajšek S., Trčak B. (2007). Mala Flora Slovenije. Ključ za Določanje Praprotnic in Semenk.

[28] Saldaña A., Gianoli E., Lusk C.H. (2005). Physiological and morphological responses to light availability in three *Blechnum* species (Pteridophyta, Blechnaceae) of different ecological breadth. Oecologia.

[29] Page C.N. (2002). Ecological strategies in fern evolution: A neopteridological overview. Rev. Palaeobot. Palynol..

[30] Cinquemani Kuehn D.M., Leopold D.J. (1993). Habitat characteristics associated with *Phyllitis scolopendrium* (L.) Newm. var. *americana* Fern. (Aspleniaceae) in central New York. Bull. Torrey Bot. Club.

[31] Vasheka O., Gratani L., Puglielli G. (2019). Frond physiological and structural plasticity of two *Asplenium* (Aspleniaceae) species coexisting in sun and shade conditions. Plant Ecol. Evol..

[32] Ok G.-H., Yoo K.-O. (2012). Habitats ecological characteristics of *Asplenium scolopendrium* L. and its RAPD analysis. Korean J. Plant Resour..

[33] Testo W.L., Watkins J.E. (2013). Understanding mechanisms of rarity in pteridophytes: Competition and climate change threaten the rare fern *Asplenium scolopendrium* var. *americanum* (Aspleniaceae). Am. J. Bot..

[34] Bremer P., Jongejans E. (2010). Frost and forest stand effects on the population dynamics of *Asplenium scolopendrium*. Popul. Ecol..

[35] Lichtenthaler H.K., Buschmann C. (2001). Extraction of photosynthetic tissues: Chlorophylls and carotenoids. Curr. Protocol. Food Anal. Chem..

[36] Lichtenthaler H.K., Buschmann C. (2001). Chlorophylls and carotenoids: Measurement and characterization by UV-VIS spectroscopy. Curr. Protocol. Food Anal. Chem..

[37] Drumm H., Mohr H. (1978). The mode of interaction between blue (UV) light photoreceptor and phytochrome in anthocyanin formation of the *Sorghum* seedling. Photochem. Photobiol..

[38] Caldwell M.M. (1968). Solar ultraviolet radiation as an ecological factor for alpine plants. Ecol. Monogr..

[39] Schreiber U., Kühl M., Klimant I., Reising H. (1996). Measurement of chlorophyll fluorescence within leaves using a modified PAM fluorometer with a fiber-optic microprobe. Photosynth. Res..

[40] Ter Braak C.J.F., Šmilauer P. (2002). CANOCO Reference Manual and CanoDraw for Windows User’s Guide: Software for Canonical Community Ordination (Version 4.5).

[41] Oliwa J., Kornas A., Skoczowski A. (2016). Morphogenesis of sporotrophophyll fronds in *Platycerium bifurcatum* depends on the red/far-red ratio in the light spectrum. Acta Physiol. Plant..

[42] Walters R.G., Horton P. (1994). Acclimation of *Arabidopsis thaliana* to the light environment: Changes in composition of the photosynthetic apparatus. Planta.

[43] Jansen M.A.K., Gaba V., Greenberg B.M. (1998). Higher plants and UV-B radiation: Balancing damage, repair and acclimation. Trends Plant Sci..

[44] Rozema J., Björn L.O., Bornman J.F., Gaberščik A., Hader D.P., Trošt T., Germ M., Klisch M., Gröniger A., Sinha R.P. (2002). The role of UV-B radiation in aquatic and terrestrial ecosystems—An experimental and functional analysis of the evolution of UV-absorbing compounds. J. Photochem. Photobiol. B.

[45] Bilger W., Rolland M., Nybakken L. (2007). UV screening in higher plants induced by low temperature in the absence of UV-B radiation. Photochem. Photobiol. Sci..

[46] Mierziak J., Kostyn K., Kulma A. (2014). Flavonoids as important molecules of plant interactions with the environment. Molecules.

[47] Potters G., Pasternak T.P., Guisez Y., Jansen M.A. (2009). Different stresses, similar morphogenic responses: Integrating a plethora of pathways. Plant Cell Environ..

[48] Mizuno M., Kyotani Y., Iinuma M., Tanaka T., Kojima H., Iwatsuki K. (1990). Kaempferol glycosides in *Asplenium scolopendrium* Newm. Z. Naturforsch. C Biosci..

[49] Iwashina T., Matsumoto S. (2011). Flavonoid properties of six *Asplenium* species in Vanuatu and New Caledonia, and distribution of flavonoid and related compounds in *Asplenium*. Bull. Natl. Mus. Nat. Sci. Ser. B.

[50] Dudek B., Warskulat A.-C., Schneider B. (2016). The occurrence of flavonoids and related compounds in flower sections of *Papaver nudicaule*. Plants.

[51] Maxwell K., Johnson G.N. (2000). Chlorophyll fluorescence—A practical guide. J. Exp. Bot..

[52] Adams W.W., Demmig-Adams B., Papageorgiou G.C. (2004). Chlorophyll fluorescence as a tool to monitor plant response to the environment. Chlorophyll a Fluorescence. A Signature of Photosynthesis. Advances in Photosynthesis and Respiration. Volume 19.

[53] Scholes J.D., Press M.C., Zipperlen S.W. (1997). Differences in light energy utilisation and dissipation between dipterocarp rainforest tree seedlings. Oecologia.

[54] Cardoso A.A., Randall J.M., McAdam S.A.M. (2019). Hydraulics regulate stomatal responses to changes in leaf water status in the fern *Athyrium filix-femina*. Plant Physiol..

[55] Hõrak H., Kollist H., Merilo E. (2017). Fern stomatal responses to ABA and CO_2_ depend on species and growth conditions. Plant Physiol..

[56] Mansfield T.A., Willmer C.M. (1969). Stomatal responses to light and carbon dioxide in the hart’s-tongue fern, *Phyllitis scolopendrium* Newm. New Phytol..

[57] Neuwirthová E., Lhotáková Z., Albrechtová J. (2017). The effect of leaf stacking on leaf reflectance and vegetation indices measured by contact probe during the season. Sensors.

[58] Gates D.M., Keegan H.J., Schleter J.C., Weidner V.R. (1965). Spectral properties of plants. Appl. Opt..

